# Expression of Interleukin-1 and Interleukin-1 Receptors Type 1 and Type 2 in Hodgkin Lymphoma

**DOI:** 10.1371/journal.pone.0138747

**Published:** 2015-09-25

**Authors:** Elisabeth Oelmann, Harald Stein, Wolfgang E. Berdel, Hermann Herbst

**Affiliations:** 1 Department of Medicine A—Hematology/Oncology, University Hospital Muenster, Muenster, Germany; 2 Pathodiagnostik Berlin, Berlin, Germany; 3 Institute of Pathology, Vivantes Hospitals, Berlin, Germany; European Institute of Oncology, ITALY

## Abstract

Signaling through the IL-1-receptor type 1 (IL-1R1), IL-1 is required for initiation and maintenance of diverse activities of the immune system. A second receptor, IL-1R2, blocks IL-1 signal transduction. We studied expression of IL-1beta, IL-1R1, and IL-1R2 in 17 Hodgkin lymphomas (HL) by *in situ* hybridization (ISH). IL-1beta expressing cells, morphologically consistent with endothelial cells and fibroblasts, occurred in all HL tissues with elevated transcript levels in areas of active fibrosis. Hodgkin and Reed-Sternberg (HRS) cells of all cases expressed low IL-1R1 transcript levels in some tumor cells, and high levels of IL-1R2 in large proportions of HRS cells. Only few bystander cells showed low levels of IL-1R1 and IL-1R2 RNA. Supernatants of 4 out of 7 HL-derived cell lines contained soluble IL-1R2 protein at high levels. HL patient sera carried variably amounts of IL-1R2 protein with significantly increased titers in patients with active disease compared to patients in complete remission and control individuals without HL. Western blots and co-immunoprecipitations showed binding of the IL-1R2 to the intracellular IL-1R-accessory protein (IL-1IRAcP). These data suggest functions of the IL-1R2 as a „decoy-receptor” sequestrating paracrine IL-1 extracellularly and intracellularly by engaging IL-1IRAcP, thus depriving IL1-R1 molecules of their extracellular and intracellular ligands. Expression of IL1-R2 by HRS cells seems to contribute to local and systemic modulation of immune function in HL.

## Introduction

Hodgkin lymphoma (HL) is characterized by a paucity of neoplastic Hodgkin- and Reed-Sternberg (HRS) cells, embedded in a variably composed reactive cellular infiltrate. HRS cells originate from B-cells [1]. Many of the distinct clinical and morphological features of HL, such as B-symptoms and the cellular composition of the reactive infiltrate, are thought to be related to a quantitatively and qualitatively abnormal expression of cytokines in HL lesions [2–5]. Some cytokines have a potential to influence immune reactions and may be responsible for the escape of HRS cells from T cell cytotoxicity [6]. This feature is particularly relevant in EBV-positive HL where HRS cells express viral *neo*-antigens known to be well controlled by T cells in healthy virus carriers [7].

The majority of lymphocytes within HL lesions belongs to the CD4+ subpopulation and is thought to have a Th2 cytokine production profile [4]. Notably in this context, HRS cells produce TGF-beta and IL-10, both of which may down modulate Th1 responses and are able to induce apoptosis of activated FAS+/CD8+ T cells and NK cells via expression of Fas-ligand (CD95L) [4]. Interleukin (IL-1)alpha and IL-1beta are pleiotropic cytokines with a broad spectrum of functions in various cell types and different tissues [8]. Both, IL-1alpha and IL-1beta have significant amino acid homology, share biologic activities, and bind to the same cell surface receptors, IL-1 receptors type 1 (IL-1R1) and type 2 (IL-1-R2) respectively [8]. Type 1 receptors mediate signaling [9] by interaction with the IL-1IRAcP, whereas type 2 receptors may be released from cells and function as decoy receptors to block IL-1beta action [10]. In addition, soluble IL-1 receptors (sIL-1R) and other naturally occurring antagonists for IL-1 such as IL-1 receptor antagonist (IL-1RA) have been described [11, 12]. Soluble IL-1R2 binds to and blocks processing of IL-1beta precursor and loses affinity for IL-1 receptor antagonist [13]. Thus, IL-1 activity seems to be the result of a finely tuned network of agonist and antagonist molecules within the IL-1 network.

IL-1, expressed by various haematopoetic and stromal cells, belongs to a group of cytokines originally described as B-cell growth factors (BCGF) [14]. IL-1 is required for lymphocyte activation in general, and has distinct functions for lymphocyte subpopulations [14–23]. One of the major co-stimulatory molecules in early T-cell activation [19–23], IL-1 prevents T-cell anergy by stimulation of IL-2 and IL-2 receptors [23–26], especially in T-helper cells [23, 27, 28]. Along with IL-12 and IL-18, IL-1 supports differentiation of Th1 cells during T-helper cell development [28]. Th2 cytokines downregulate IL-1 expression, whereas the antagonizing molecule IL-1RA is up-regulated by Th2 cells [28, 29]. In concert with IL-2, IL-1 augments the activating function of IL-2 on lymphokine activated killer cell (LAK)–activity [30–32], and enhances the function of cytotoxic CD4 -, CD8—and NK cells in concert with IL-2 and interferons. IL-1 functions hereby as an enhancer of immunological defense mechanisms against malignancies [33, 34]. Taken together IL-1 is involved in initiating and maintaining optimal activated conditions of the immune system, preventing anergy and inducing elimination of tumor cells. Regarding the direct effects of IL-1 on tumor cells, stimulation [35–37] as well as inhibition [38–42] of growth has been observed with different tumor cells.

We studied IL-1 and its receptors in HL tissue sections, the presence of IL-1R2 in plasma of HL patients, and the synthesis of these proteins by HL derived cell lines (HLDCL). Data suggest a paracrine function of IL-1beta in HL lesions and a modulatory role of IL-1R2 as a specific product of HRS cells likely to inhibit possible differentiating functions of IL-1 on the tumor cell itself, and to induce anergy in the surrounding lymphoid tissue.

## Patients and Methods

### Patients

Lymph nodes were from 13 patients with nodular sclerosis (NSHL) and 10 patients with mixed cellularity (MCHL) type of HL submitted for histopathological diagnosis prior to treatment. Tissues were routinely formalin-fixed and paraffin-embedded. Slides from stored material were grouped into NSHL or MCHL and otherwise completely anonymized. Heparin-plasma from 20 patients treated in our department within the studies of the German Hodgkin Lymphoma Study Group were available to ELISA studies with HL in complete remission (CR) and 18 patients with active disease present (DP; NSHL or MCHL, no further selection). Plasma was stored frozen. Aliquots were grouped for CR or DP, otherwise completely anonymized, and directly taken for ELISA analysis. Control plasma was from healthy individuals. Material was destroyed after usage. This study was approved by the Ethical Board of the Faculty for Medicine of the Westfaelische Wilhelms-Universitaet Muenster and the Physician´s Chamber of Westfalen-Lippe (2015-174-f-S). Since old anonymized storage material was used, exemption from written informed consent was granted.

### HL derived cell lines

Hodgkin-Reed/Sternberg (H-R/S) cell derived cell lines (HLDCL) were kindly provided by Prof. Volker Diehl (Univ. of Cologne, Germany), by Dr. D. B. Jones (Southampton, UK), and Dr. H. Kamesaki (Kyoto, Japan). Morphology and immunophenotype of the cell lines have been described [43]. Cell lines were cultured in AIMV-V medium (Gibco BRL, Gaithersburg, MD, USA) without fetal calf serum (FCS). Cell lines repeatedly tested negative for mycoplasma infection during the experiments.

### *In situ* hybridization (ISH)

After linearisation of plasmids (pGEM-3Z, Promega, Madison, Wisconsin, USA) containing specific sequences of the genes for hIL-1beta (R&D Systems, Minneapolis, USA) and hIL-1R type 1 and type 2 (kindly provided by Immunex, Seattle, WA, USA), 35S-labeled run-off anti-sense and sense (control-) transcripts were generated using Sp6 and T7 RNA polymerases (Gibco BRL). ISH for the detection of RNA transcripts was performed as previously described [2]. In brief, dewaxed and rehydrated paraffin sections were exposed to 0.2 N HCL and 0.125 mg/ml pronase (Boehringer, Mannheim, Germany) followed by acetylation with 0.1 M triethanolamine pH 8.0/0.25% (v/v) acetic anhydride and dehydration through graded ethanols. Slides were hybridized to 2–4 x 10^5^ cpm of labeled probes overnight at 54°C. Washing and autoradiography was performed as described [2]. All sections were processed in parallel using the same batches of reagents and probes. The incubation of sections with *Micrococcus* nuclease (Boehringer Mannheim, Mannheim, Germany) prior to in situ hybridization resulted in the extinction of the specific autoradiographic signal, establishing that RNA sequences were the targets of the hybridization procedure. ISH signals were semiquantitated by counting the proportion of positive HRS cells and estimating the density of silver grains as the correlate for the transcript levels.

### Enzyme-linked immunosorbant assay (ELISA)

IL-1R2 plasma levels and levels in HLDCL supernatants were measured by ELISA kits (R+D Systems, Wiesbaden, Germany) as described by the manufacturer. Plasma (stored at –80°C) was measured either directly or after further dilution. Cells from cell lines were washed and cultured at 10^6^ cells per 20 ml of AIM-V medium for 48 hrs (pH 7.2, 37°C, 5% CO_2_ and high humidity). Subsequently, culture supernatants were harvested, stored at –80°C and directly taken for ELISA or assayed after further dilution.

### Western blot, immunoprecipitation

Western blot and Immunoprecipitation were carried out according standard procedures. In brief, cells from KMH2 (2 x 10^7^ cells/300 microliter) were lysed with Special Lysis Buffer (20mM Tris, pH 7.4, 1mM EGTA, 1mM EDTA, 2mM DTT, 0.5% TritonX-100) on ice, incubated for 20 minutes at 4°C, centrifuged at 13000rpm at 4°C, and supernatants were stored at –80°C. For Western blot 40 microliter of the lysate was boiled with 3 x SDS buffer for 5 minutes and then transferred directly into a 4–15% ready to use gel (Bio-Rad Laboratories, München). For immunoprecipitation the lysates were first incubated with first antibodies and Sepharose-G-beats over night and then centrifuged at 2500 rpm for 1 minute. The pellet was 3 x times washed with Special Lysis Buffer, boiled with 3 x SDS, centrifuged at 2500 rpm for 1 minute, and 20 microliter of the supernatant was transferred to the gel. The gel was running 1h 30 minutes and then transferred to a nitrocellulose membrane (Hybond ECL, Amersham Biosciences Europe, Freiburg) for 3 h. The membrane was then blocked with 5% dry milk (Fluka Chemie, Deisenhofen), incubated with the second antibody solution and washed with 1x PBS. Detection was carried out with HRP conjugated antibodies and Luminol reagent (Santa Cruz Biotechnology Inc., Heidelberg).

### Statistics

Results were evaluated statistically by the Mann-Whitney test. P-values < .05 were interpreted as indicating significant differences.

## Results

### IL-1beta RNA expression in HL tissue sections

**Fig 1:**
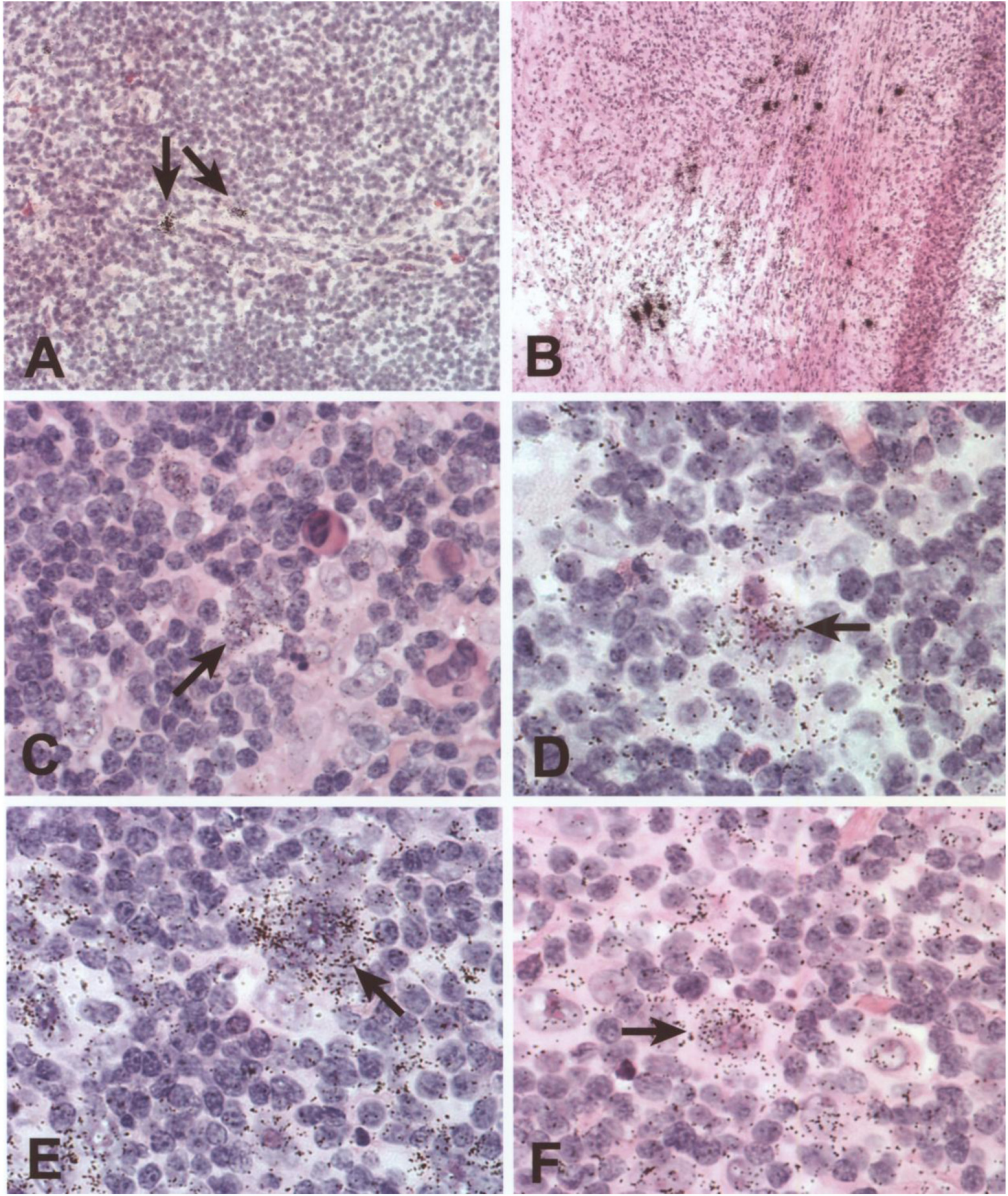
IL-1beta, IL-1R1, IL-1R2 expression in lymph node sections of patients with Hodgkin’s disease as observed with *in situ* Hybridization (ISH) (Fig 1A–1F). Positive cells are characterized by accumulation of black stains (examples illustrated with arrows). **A)** IL-1beta expression in few stromal and endothelial cells throughout the lymph node of MC type HL. **B)** IL-1beta expression in the stromal cells of areas with tissue remodeling and sclerosis in NSHL. **C, D)** IL-1R1 expression in HRS cells and few positive lymphocytes in MCHL **(C)**, and NSHL **(D)**. **E, F)** IL-1R2 positive HRS cells in a case of MCHL (E) and NSHL **(F)**.

**Fig 2:**
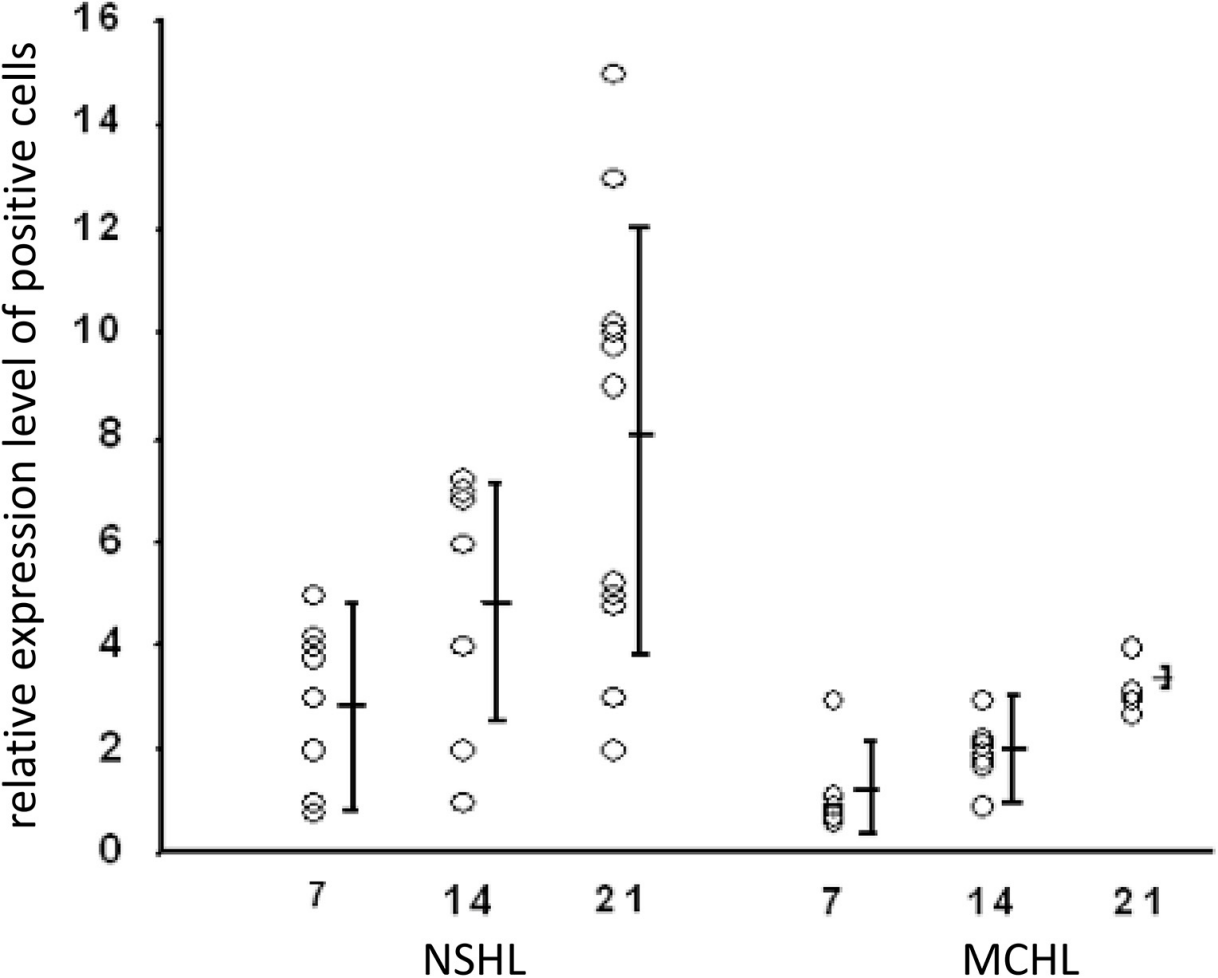
IL-1beta expression in HRS cells as observed by ISH after 7, 14, and 21 days of autoradiography. MCHL, mixed cellularity (n = 6). NSHL, nodular sclerosis (n = 11). Each circle represents 1 individual patient. Y-axis shows the relative expression level of positive cells, which comprises the number of grains per cell and the expression intensity throughout the slide, with mean values and standard deviation. There was a statistically significant difference between the histological subtypes after 21 days (p = .042, Mann-Whitney test).

IL-1beta expression was detected in all sections of 6 and 11 lymph nodes with MCHL and NSHL, respectively (**Fig 1A and 1B**). The specific autoradiographic signal was most intense in stromal cells of areas with active tissue remodeling and sclerosis (**Fig 1B**). Accordingly, there was a significant difference between NSHL and MCHL with high transcript levels of IL-1beta in NSHL (**Fig 2**). The expression pattern in MCHL was restricted to few stromal cells, morphologically compatible with fibroblasts and endothelial cells, in scattered distribution throughout the lesion (**Fig 1A**). In all HL cases, IL-1beta was absent from HRS cells at levels detectable by our in situ hybridization technique.

### Human IL-1 receptor type 1 and type 2 transcripts in HL tissue sections

Transcripts from both receptor genes were detectable in all of 17 HL cases (**Fig 1C, 1D, 1E and 1F**). HRS cells showed specific autoradiographic signals for both receptors, and particularly elevated levels of IL-1R2 transcripts (**Fig 1E**). The signal intensity and the proportion of specifically labeled HRS cells varied between patients, with a range between 5% and 95% percent of HRS cells per case within one tissue. IL-1R2 transcript levels in HRS cells were higher than IL-1R1 transcript levels. The reactive lymphoid infiltrate showed irregularly distributed cells with weak expression of the IL-1R1 (**Fig 1D**), whereas IL-1R2-positive reactive cells were particularly rare (**Fig 1E**).

### ELISA for IL-1R2 protein levels in supernatants from HLDCL

**Fig 3:**
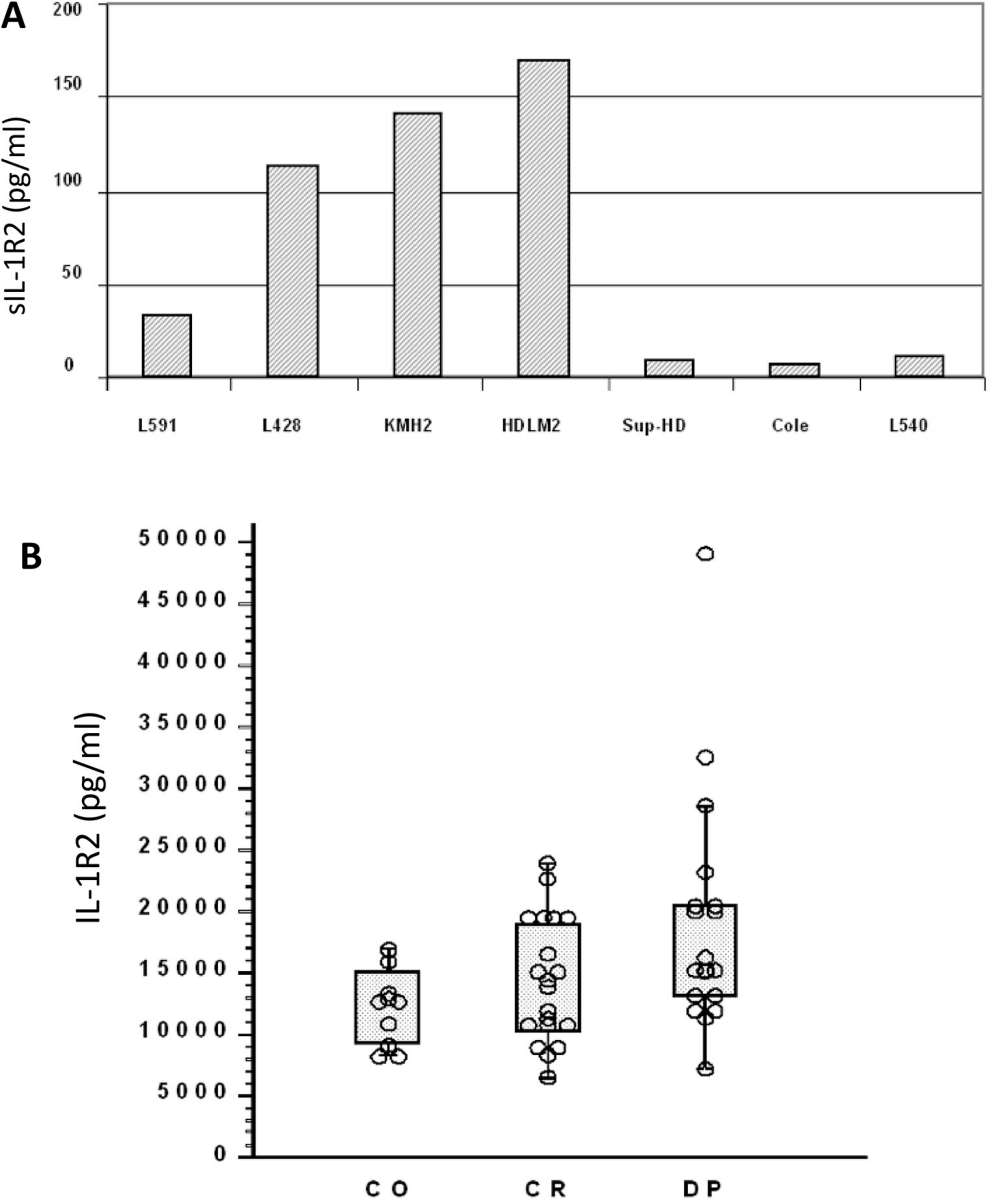
IL-1R2 concentrations in supernatants and plasma. **A)** IL-1R2 concentration (pg/ml) in supernatants of HL derived cell lines as measured by ELISA (for details see Materials and Methods). **B)** IL-1R2 concentration in plasma of patients with Hodgkin’s disease (CR, complete remission; DP, active disease present) in comparison to control persons (CO) as measured by ELISA (for details see Patients and Methods). Each circle represents 1 individual person. Statistical comparison (Mann-Whitney test) revealed significant differences when values were compared as follows: DP vs. CR (p = .037), DP vs. CO (p = .018), CR vs. CO (p = .34).

The cell lines, L591, L428, KMH2, and HDLM2 produced soluble IL-1R2 protein at concentrations between 30 and 170 pg/ml in their supernatants (**Fig 3A**). Experiments were reproduced twice with only little variation. Three of the cell lines, Sup-HD, Cole and L540 showed hIL-1R2 levels below the detection level and were thus considered non-producers.

### ELISA for IL-1R2 protein in sera of HL patients and healthy controls

Sera from patients diagnosed with HL disease present (DP; n = 18), or in complete remission (CR; n = 20) were studied for the presence of hIL-1R2 protein in comparison to normal individuals (CO; n = 10) (**Fig 3B**). Sera of all HL patients presented with variable levels of IL-1R2. However, patients with active disease (progression of disease; DP) showed significantly higher levels compared to patients in complete remission (CR) and healthy individuals (controls; CR). IL-1R2 serum levels of normal controls were similar to those of HL patients in CR.

### Co-Immunoprecipitation of IL-1R2 and the IL-1 receptor accessory protein IL-1IRAcP

**Fig 4:**
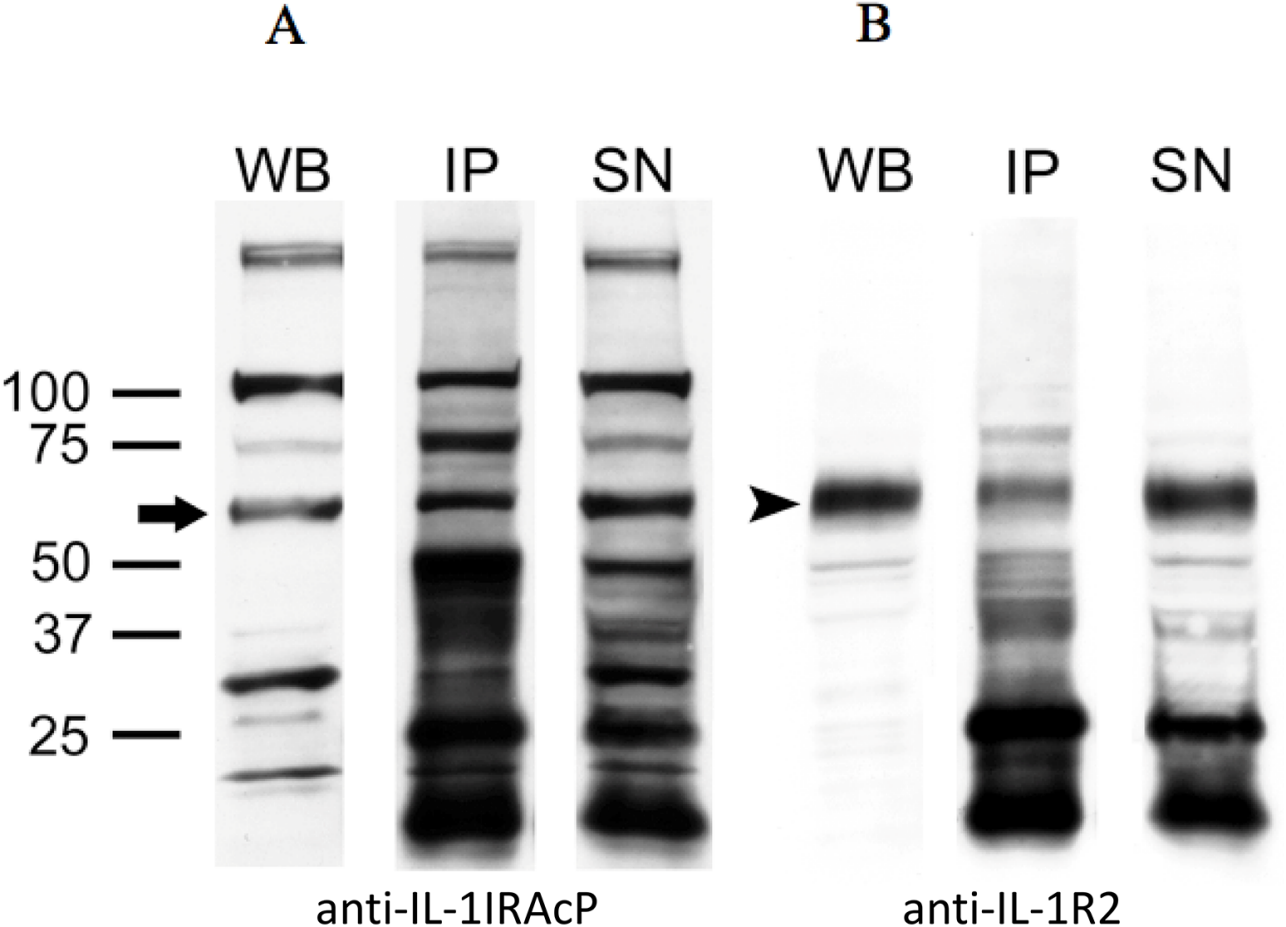
A) Western blot (WB), immunoprecipitation (IP), and supernatants (SN) of the immunoprecipitation reaction with anti-IL-1IRAcP is shown in the left panel (Fig 4A). Westernblot of whole lysates of KMH2 is shown in the left lane (WB), the immunoprecipitated IL-1IRAcP is shown in the middle lane (IP), and the supernatant of this IP-reaction (SN), is shown in the right lane. The blot was detected also with the same antibody, anti-IL-1IRAcP, as a control reaction for the IP-reaction. The band in the supernatant indicates, that there was too much protein to get all immunoprecipitated, so that the amount of IL-1IRAcP, which was not bound by the antibody, occurs in the supernatant. IL-1IRAcP positive band is shown at the expected size of 66kDa (black arrow). Y-axis, size markers. **B)** After stripping the blot, detection was carried out with a specific anti-IL-1R2 antibody. Reactivity of the antibody was shown in the WB-, IP- and SN-lanes of KMH2, showing that the cell line expresses high amounts of membrane bound IL-1R2 (WB), which in part is able to interact with IL-1IRAcP (IP).

Lysates from the IL-1R2 high producing cell line, KMH2, were studied to characterize a possible interaction of IL-1R2 with the IL-1IRAcP. Immunopreciptitation was carried out by incubating the KMH2-lysates with anti-IL-1IRAcP and subsequent characterization of the precipitate again with anti-IL-1IRAcP (**Fig 4, left panel**) to show that the immunoprecipitation was successful. The Blot was then stripped and developed again with anti-IL-1R2 (**Fig 4, right panel**). The bands observed, correspond to the established sizes of IL-1IRAcP and IL-1R2, of 66kDa and 68kDa respectively, verifying that IL-1IRAcP interact with IL-1R2 in KMH2. We can not make any statement about the quantity of interacting receptors *in vivo*, because depending on the effectiveness of the IP-reaction, anti-IL-1IRAcP is unlikely to absorb all of the protein. This may explain why the supernatants display bands of both, IL-1IRAcP (**Fig 4A, SN**) and IL-1R2 (**Fig 4B, SN**).

## Discussion

Exploring the potential role of the IL-1 network in HL, we studied expression of IL-1beta, IL-1R1, and IL-1R2. The results suggest a paracrine role for IL-1beta in HL and expression and shedding of IL-1R2 as a mechanism by which HRS cells may modulate IL-1 activity intracellularly and in their microenvironement.

Our finding of IL-1beta RNA expression in HL tissues confirms and extends previous reports on IL-1alpha and IL-1beta expression. IL-1beta expressing cells were more numerous in areas of active fibrosis in NSHL. IL-1beta has a fibrogenic effect, as shown by Postlethwaite et al. [44] and this may contribute to the formal pathogenesis of NSHL, in particular. Unlike many other cytokines and growth factors, IL-1beta is not expressed by HRS cells at levels that are detectable by our sensitive *in situ* hybridization technique. When activated by IL-1 converting enzyme, IL-1alpha and IL-1beta may bind to the IL-1R1 [8]. Signaling through this receptor requires the presence of IL-1IRAcP to form an intracellular complex [9]. Expression of IL-1R1 is typically found on normal B cells, hence the previous characterization of IL-1 as B-cell growth factor, BCGF [14]. HRS cells were characterized as originating from B lymphocytes. Therefore, IL-1R1 expression in some HRS cells may be reminiscent of that lineage.

Expression of IL-1R1, however, has to be viewed in the context of IL-1R2 expression, which, according to our *in situ* hybridization patterns, was present in stronger signal intensities and in larger proportions of tumor cells than expression of IL-1R1. IL-1R2 has functions as a ligand sink through 3 mechanisms: membrane bound IL-1R2 interferes with IL-1 signaling by sequestration of the accessory protein, IL-1IRAcP, thus depriving the IL-1R1 from its intracellular signaling partner [45–47], sIL-1R2 blocks processing of IL-1beta precursors [12], and the soluble complex of IL-1R2 and IL-1IRAcP has an approximately 100-fold increased affinity for IL-1 compared to sIL-1R2 alone [48]. Moreover, the latter complex does not bind IL-1RA, so that these molecules are additionally available to antagonize IL-1R1. According to our data, it is conceivable that all of these mechanisms may be functional in HL.

Most HRS cells in all of our cases expressed the IL-1R2 at significantly higher levels, and the presence of sIL-1R2 in HLDCL supernatants and in HL patient sera suggest that the RNA is translated to protein. Although we cannot exclude other sources of IL-1R2, we found high amounts of IL-1R2 RNA almost exclusively in HRS cells, so that high IL-1R2 serum levels most likely originate from these cells.

Counteracting the effects of IL-1 may be advantageous to HRS cells in their microenvironment. In this context, its direct actions on tumor cells, either growth stimulatory [35–37, 49] or inhibitory [38–42], and the modulation of tumor specific cytotoxic CD4-, CD8- [30], LAK- [31, 32] and NK-cells [50] are of interest and should be further studied.

The diversity of direct effects of IL-1 on tumor cells may be related to the differentiation status of cells and also to the type of assay, most of which do not discriminate between proliferation as self renewal or as transition to the terminal stage of differentiation, as described for IL-1 function on glioblastomas [51]. In general, IL-1 functions involve signal transduction pathways of GTP-binding proteins, such as sphingomyelinases, PKC, phospholipases, casein kinases, and result in activation of MAP-kinase-, IκB/NFκB-, and c-Jun N-terminal kinase (JNK) family members with subsequent transcription of genes such as by NFκB and AP-1 transcription factors [52]. In B-cells, these Toll/IL-1 receptor pathways seem to be responsible for IL-1 effects, such as differentiation/proliferation and antibody synthesis [53–56], both of which may be inhibited in HRS cells by sequestering IL-1IRAcP by IL-1R2.

In view of the extensive infiltration of HL lesions by cells of the immunosurveillance system, immune escape mechanisms are of paramount importance to explain the existence of HRS cells, in particular when expressing viral neo-antigens such as LMP1 in EBV-associated cases. Previously, expression of IL-10, TIMP-1, TARC and other factors have been described with immunomodulatory functions, and IL-1R2 expression seems to be an additional mechanism counteracting immune activation. Expression of IL-1R2 seems to be one of the „road blocks” [57] employed by HRS cells to interfere with normal immune functions of the surrounding lymphocytes and to counteract IL-1 action on the tumor cells themselves.

Expression of IL-1R2 was originally found in Raji cells, an EBV-positive B-cell line [58, 59], and IL-1R2 has sequence homology of approx. 30% to the Vaccinia virus gene B15R [60, 61]. Therefore, IL-1R2 expression may be regarded as a general mechanism of escape from IL-1 induced, adverse immune effects. In viral infections down modulation of IL-1 effects have two aspects, protection of the virus-infected cell itself and protection of the host from excessive inflammation [60]. Several lines of evidence indicate that the immunological function of IL-1 in Hodgkin’s disease is similarly inhibited, which may be partially explained by high amounts of soluble IL-1R2.

Although IL-1 mediated effects were considered responsible for B-symptoms in progressive Hodgkin’s disease, IL-1alpha and beta were, when detectable, present in only small amounts in patients sera [62]. Presence of IL-1RA, as shown in some sera of Hodgkin patients [63], may only partially contribute for this effect, because of its inability to bind the cytokine itself. Our data show a significant correlation between sIL-1R2 expression by HRS-cells and the activity of disease. The physiological function of sIL-1R2, to capture IL-1beta from the system, is therefore enhanced in patients with progressive disease and could be the reason for only small amounts of IL-1beta to be found in sera of Hodgkin’s patients. From the high levels of IL-1R2 RNA and protein expression by HRS cells one can assume that the surrounding lymphoid tissue, neighboring the tumor cells, is rendered unresponsive by inhibition of IL-1 dependent activation.

IL-1 is known as one main inducer of IL-2 responsiveness in T-cells, a function severely impaired in HL: lymphocytes prepared from HD lesions have been shown to be defective in producing IL-2 and IL-2 receptors with decreased proliferative response to PHA, with reduced blastogenesis and T-cell colony formation [64–67]. Expression of IL-2 and IL-2 receptors in T-cells from HL tissues is severely impaired [64, 66], moreover the proliferative response of normal PBMNCs is also severely inhibited, when cultivated with sera of Hodgkin’s patients [64]. Furthermore, blocking IL-1 function involves inhibition of IL-1 dependent signal transduction pathways described above, probably also in the surrounding lymphocytes which would result in inhibition of IL-1 dependent function in B-cells (differentiation/proliferation and antibody synthesis) [53–56], in T-helper cells (differentiation towards Th1 cells) [28], in CD8+ T-cells (antigen-specific cross-priming) [68–70], and overall inhibition of stimulation of CD4/CD8 [17], NK [50, 69], gamma-delta T-cells [70] and LAK cells [31, 32].

In summary, we confirm earlier reports on expression of IL-1 in Hodgkin tissue [71–73] and report for the first time on IL-1R2 transcripts and protein abundantly present in HRS cells, which probably counteract IL-1 function systemically and in the microenvironment of the Hodgkin lesion. IL-1R2 thereby may contribute to the spectrum of tumor induced immune escape mechanisms by inhibition of IL-1, one of the earliest general T-cell co-stimulatory molecules and by deprivation of HRS cells from regulation by IL-1. This observation may contribute to understanding the complex signaling network within the Hodgkin lesion microenvironment [73–75] and may help to explore new immune-therapy approaches for resistant cases [76].

## References

[1] Jungnickel B, Staratschek-Jox A, Brauninger A, Spieker T, Wolf J, Diehl V, et al Clonal deleterious mutations in the IkappaBalpha gene in the malignant cells in Hodgkin's lymphoma. J Exp Med 2000;191(2): 395–402. 10637284 10.1084/jem.191.2.395 PMC2195754

[2] Foss HD, Herbst H, Oelmann E, Samol J, Grebe M, Blankenstein T, et al Lymphotoxin, tumour necrosis factor and interleukin-6 gene transcripts are present in Hodgkin and Reed-Sternberg cells of most Hodgkin's disease cases. Br J Haematol 1993;84: 627–635. 8217820 10.1111/j.1365-2141.1993.tb03138.x

[3] Foss HD, Hummel M, Gottstein S, Ziemann K, Falini B, Herbst H, et al Frequent expression of IL-7 gene transcripts in tumor cells of classical Hodgkin's disease. Am J Pathol 1995;146: 33–39. 7856736 PMC1870751

[4] Poppema S, Potters M, Visser L, van den Berg AM. Immune escape mechanisms in Hodgkin's disease. Ann Oncol 1998;9 Suppl 5: S21–24. 9926233 10.1093/annonc/9.suppl_5.s21

[5] Jundt F, Anagnostopoulos I, Bommert K, Emmerich F, Muller G, Foss HD, et al Hodgkin/Reed-Sternberg cells induce fibroblasts to secrete eotaxin, a potent chemoattractant for T cells and eosinophils. Blood 1999;94: 2065–2071. 10477736

[6] Gabrilovich D, Pisarev V. Tumor Escape from Immune Response: Mechanisms and Targets of Activity. Current Drug Targets 2003;4: 525–536. 14535653 10.2174/1389450033490849

[7] Steigerwald-Mullen P, Kurilla MG, Braciale TJ. Type 2 cytokines predominate in the human CD4(+) T-lymphocyte response to Epstein-Barr virus nuclear antigen 1. J Virol 2000;74: 6748–6759. 10888613 10.1128/jvi.74.15.6748-6759.2000 PMC112191

[8] Dinarello CA.The biological properties of interleukin-1. Eur Cytokine Netw 1994;5: 517–531. 7727685

[9] Sims JE, Gayle MA, Slack JL, Alderson MR, Bird TA, Giri JG, et al Interleukin 1 signaling occurs exclusively via the type I receptor. Proc Natl Acad Sci U S A 1993;90: 6155–6159. 8327496 10.1073/pnas.90.13.6155 PMC46886

[10] Colotta F, Re F, Muzio M, Bertini R, Polentarutti N, Sironi M, et al Interleukin-1 type II receptor: a decoy target for IL-1 that is regulated by IL-4. Science 1993;261: 472–475. 8332913 10.1126/science.8332913

[11] Arend WP. Interleukin-1 receptor antagonist. Adv Immunol 1993;54: 167–227. 8379462 10.1016/s0065-2776(08)60535-0

[12] Larrick JW. Native interleukin 1 inhibitors. Immunol Today 1989;10: 61–66. 2526643 10.1016/0167-5699(89)90308-3

[13] Symons JA, Young PR, Duff GW. Soluble type II interleukin 1 (IL-1) receptor binds and blocks processing of IL-1 beta precursor and loses affinity for IL-1 receptor antagonist. Proc Natl Acad Sci U S A 1995;92: 1714–1718. 7878046 10.1073/pnas.92.5.1714 PMC42590

[14] Gordon J, Cairns JA. Autocrine regulation of normal and malignant B lymphocytes. Adv Cancer Res 1991;56: 313–334. 1851375 10.1016/s0065-230x(08)60484-4

[15] Matsushima K, Taguchi M, Kovacs EJ, Young HA, Oppenheim JJ. Intracellular localization of human monocyte associated interleukin 1 (IL 1) activity and release of biologically active IL 1 from monocytes by trypsin and plasmin. J Immunol 1986;136: 2883–2891. 2420874

[16] Dinarello CA. Interleukin-1 and its biologically related cytokines. Adv Immunol 1989;44: 153–205. 2466396 10.1016/s0065-2776(08)60642-2

[17] Numerof RP, Kotik AN, Dinarello CA, Mier JW. Pro-interleukin-1 beta production by a subpopulation of human T cells, but not NK cells, in response to interleukin-2. Cell Immunol. 1990;130: 118–128. 2144468 10.1016/0008-8749(90)90166-o

[18] Carosella ED, Tilden AB, Dunlap NE. Human B cell differentiation by Fc fragment III. Effect of IL-1 and IL-2 on differentiation of human B lymphocytes induced by Fc fragments of human IgG. Cell Immunol. 1989;121: 269–279. 2472222 10.1016/0008-8749(89)90025-7

[19] Tosato G, Miller J, Marti G, Pike SE. Accessory function of interleukin-1 and interleukin-6: preferential costimulation of T4 positive lymphocytes. Blood. 1990;75: 922–930. 1967955

[20] Rothenberg EV, Diamond RA. Costimulation by interleukin-1 of multiple activation responses in a developmentally restricted subset of immature thymocytes. Eur J Immunol. 1994;24: 24–33. 8020563 10.1002/eji.1830240105

[21] Stricker K, Serfling E, Krammer PH, Falk W. An NF-kappa B-like element plays an essential role in interleukin-1-mediated costimulation of the mouse interleukin-2 promoter. Eur J Immunol. 1993;23: 1475–1480. 8325323 10.1002/eji.1830230712

[22] Briscoe DM, Henault LE, Geehan C, Alexander SI, Lichtman AH. Human endothelial cell costimulation of T cell IFN-gamma production. J Immunol. 1997;159: 3247–3256. 9317123

[23] Conti P, Panara MR, Porrini AM, Gambi D, Barbacane RC, Reale M, et al Inhibition of interleukin-1 (alpha and beta), interleukin-2 secretion and surface expression of interleukin-2 receptor (IL-2R) by a novel cytokine interleukin-1 receptor antagonist (IL-1ra). Scand J Immunol. 1992;36: 27–33. 1535451 10.1111/j.1365-3083.1992.tb02937.x

[24] Ales-Martinez JE, Cuende E, Gaur A, Scott DW. Prevention of B cell clonal deletion and anergy by activated T cells and their lymphokines. Semin Immunol. 1992;4: 195–202. 1627790

[25] Hudspith BN, Brostoff J, McNicol MW, Johnson NM. Anergy in sarcoidosis: the role of interleukin-1 and prostaglandins in the depressed in vitro lymphocyte response. Clin Exp Immunol. 1984;57: 324–330. 6331921 PMC1536124

[26] Bourdoulous S, Beraud E, Le Page C, Zamora A, Ferry A, Bernard D, et al Anergy induction in encephalitogenic T cells by brain microvessel endothelial cells is inhibited by interleukin-1. Eur J Immunol. 1995;25: 1176–1183. 7539749 10.1002/eji.1830250507

[27] Williams JM, Ransil BJ, Shapiro HM, Strom TB. Accessory cell requirement for activation antigen expression and cell cycle progression by human T lymphocytes. J Immunol. 1984;133: 2986–2995. 6092462

[28] Heath VL, Kurata H, Lee HJ, Arai N, O'Garra A. Checkpoints in the regulation of T helper 1 responses. Curr Top Microbiol Immunol. 2002;266: 23–39. 12014201 10.1007/978-3-662-04700-2_3

[29] Kovalovsky D, Refojo D, Holsboer F, Arzt E. Molecular mechanisms and Th1/Th2 pathways in corticosteroid regulation of cytokine production. J Neuroimmunol. 2002;109: 23–29. 10.1016/s0165-5728(00)00298-8 10969177

[30] Ebina N, Gallardo D, Shau H, Golub SH. IL-1 and IL-4 as reciprocal regulators of IL-2 induced lymphocyte cytotoxicity. Br J Cancer. 1990;62: 619–623. 1699593 10.1038/bjc.1990.341 PMC1971504

[31] Crump WL, Owen-Schaub LB, Grimm EA. Synergy of human recombinant interleukin 1 with interleukin 2 in the generation of lymphokine-activated killer cells. Cancer Res. 1989;49: 149–153. 2783241

[32] Fujiwara T, Grimm EA. Regulation of lymphokine-activated killer cell induction by human recombinant IL-1 receptor antagonist. Obligate paracrine pathway of IL-1 during lymphokine-activated killer cell induction. J Immunol. 1992;148: 2941–2946. 1374105

[33] Wang SY, Hsu ML, Hsu HC, Tzeng CH, Lee SS, Shiao MS, et al The anti-tumor effect of Ganoderma lucidum is mediated by cytokines released from activated macrophages and T lymphocytes. Int J Cancer. 1997;70: 699–705. 9096652 10.1002/(sici)1097-0215(19970317)70:6<699::aid-ijc12>3.0.co;2-5

[34] Saijo Y, Hong X, Tanaka M, Tazawa R, Liu SQ, Saijo K, et al Autologous high-killing cytotoxic T lymphocytes against human lung cancer are induced using interleukin (IL)-1beta, IL-2, IL-4, and IL-6: possible involvement of dendritic cells. Clin Cancer Res. 1999;5:1203–1209. 10353758

[35] Lahm H, Petral-Malec D, Yilmaz-Ceyhan A, Fischer JR, Lorenzoni M, Givel JC, et al Growth stimulation of a human colorectal carcinoma cell line by interleukin-1 and -6 and antagonistic effects of transforming growth factor beta 1. Eur J Cancer. 1992;28A: 1894–1899. 1389533 10.1016/0959-8049(92)90031-v

[36] Hamburger AW, Lurie KA, Condon ME. Stimulation of anchorage-independent growth of human tumor cells by interleukin 1. Cancer Res. 1987;47: 5612–5615. 3499215

[37] Zeki K, Nakano Y, Inokuchi N, Watanabe K, Morimoto I, Yamashita U, et al Autocrine stimulation of interleukin-1 in the growth of human thyroid carcinoma cell line NIM 1. J Clin Endocrinol Metab. 1993;76: 127–133. 8421076 10.1210/jcem.76.1.8421076

[38] Onozaki K, Matsushima K, Aggarwal BB, Oppenheim JJ. Human interleukin 1 is a cytocidal factor for several tumor cell lines. J Immunol. 1985;135: 3962–3968. 2415593

[39] Lachman LB, Dinarello CA, Llansa ND, Fidler IJ. Natural and recombinant human interleukin 1-beta is cytotoxic for human melanoma cells. J Immunol. 1986;136:3098–3102. 3485680

[40] Bertoglio JH, Rimsky L, Kleinerman ES, Lachman LB. B-cell line-derived interleukin 1 is cytotoxic for melanoma cells and promotes the proliferation of an astrocytoma cell line. Lymphokine Res. 1987;6: 83–91. 3495709

[41] Danforth DN Jr., Sgagias MK. Interleukin-1 alpha and interleukin-6 act additively to inhibit growth of MCF-7 breast cancer cells in vitro. Cancer Res. 1993;53: 1538–1545. 8453620

[42] Kilian PL, Kaffka KL, Biondi DA, Lipman JM, Benjamin WR, Feldman D, et al Antiproliferative effect of interleukin-1 on human ovarian carcinoma cell line (NIH:OVCAR-3). Cancer Res. 1991;51: 1823–1828. 1825935

[43] Drexler H. Recent results on the biology of Hodgkin and Reed-Sternberg cells. II. Continuous cell lines. Leukemia Lymphoma. 1993;9: 1–25. 7682880 10.3109/10428199309148499

[44] Postlethwaite AE, Raghow R, Stricklin GP, Poppleton H, Seyer JM, Kang AH. Modulation of fibroblast functions by IL-1: increased steady-state accumulation of type I procollagen messenger RNAs and stimulation of other functions but not chemotaxis by human recombinant interleukin 1 alpha and beta. J Cell Biol. 1988;106: 311–318. 2828381 10.1083/jcb.106.2.311 PMC2114989

[45] Lang D, Knop J, Wesche H, Raffetseder U, Kurrle R, Boraschi D, et al The type II IL-1 receptor interacts with the IL-1 receptor accessory protein: a novel mechanism of regulation of IL-1 responsiveness. J Immunol. 1998;161: 6871–6877. 9862719

[46] Malinowsky D, Lundkvist J, Laye S, Bartfai T. Interleukin-1 receptor accessory protein interacts with the type II interleukin-1 receptor. FEBS Lett. 1998;429: 299–302. 9662436 10.1016/s0014-5793(98)00467-0

[47] Neumann D, Kollewe C, Martin MU, Boraschi D. The membrane form of the type II IL-1 receptor accounts for inhibitory function. J Immunol. 2000;165: 3350–3357. 10975853 10.4049/jimmunol.165.6.3350

[48] Smith DE, Hanna R, Della F, Moore H, Chen H, Farese AM, et al The soluble form of IL-1 receptor accessory protein enhances the ability of soluble type II IL-1 receptor to inhibit IL-1 action. Immunity. 1993;18: 87–96. 10.1016/s1074-7613(02)00514-9 12530978

[49] Ito R, Kitadai Y, Kyo E, Yokozaki H, Yasui W, Yamashita U, et al Interleukin 1 alpha acts as an autocrine growth stimulator for human gastric carcinoma cells. Cancer Res. 1993;53: 4102–4106. 8358739

[50] Naume B, Espevik T. Immunoregulatory effects of cytokines on natural killer cells. Scand J Immunol. 1994;40: 128–134. 8047834 10.1111/j.1365-3083.1994.tb03441.x

[51] Oelmann E, Kraemer A, Serve H, Reufi B, Oberberg D, Patt S, et al Autocrine interleukin-1 receptor antagonist can support malignant growth of glioblastoma by blocking growth-inhibiting autocrine loop of interleukin-1. Int J Cancer. 1997;71: 1066–1076. 9185713 10.1002/(sici)1097-0215(19970611)71:6<1066::aid-ijc25>3.0.co;2-a

[52] Martin MU, Wesche H. Summary and comparison of the signaling mechanisms of the Toll/interleukin-1 receptor family. Biochim Biophys Acta. 2002;1592: 265–280. 12421671 10.1016/s0167-4889(02)00320-8

[53] Iwasaki T, Sims JE, Grabstein K, Dower SK, Rachie N, Bomsztyk K. Comparison of IL-1 alpha effectiveness in activating murine pre-B and T cell lines. Cytokine. 1993;5: 416–426. 8142596 10.1016/1043-4666(93)90031-y

[54] Bomsztyk K, Toivola B, Emery DW, Rooney JW, Dower SK, Rachie NA, et al Role of cAMP in interleukin-1-induced kappa light chain gene expression in murine B cell line. J Biol Chem. 1990;265: 9413–9417. 2160978

[55] Nakae S, Asano M, Horai R, Sakaguchi N, Iwakura Y. IL-1 enhances T cell-dependent antibody production through induction of CD40 ligand and OX40 on T cells. J Immunol. 2001;167: 90–97. 11418636 10.4049/jimmunol.167.1.90

[56] Suzuki N, Suzuki S, Duncan GS, Millar DG, Wada T, Mirtsos C, et al Severe impairment of interleukin-1 and Toll-like receptor signaling in mice lacking IRAK-4. Nature. 2002;416: 750–756. 11923871 10.1038/nature736

[57] Dinarello CA. (1998) Interleukin-1 beta, interleukin-18, and the interleukin-1 beta converting enzyme. Ann N Y Acad Sci. 1998;856: 1–11. 9917859 10.1111/j.1749-6632.1998.tb08307.x

[58] Giri JG, Newton RC, Horuk R. Identification of soluble interleukin-1 binding protein in cell-free supernatants. Evidence for soluble interleukin-1 receptor. J Biol Chem. 1990;265: 17416–17419. 2145273

[59] Symons JA, Duff GW. A soluble form of the interleukin-1 receptor produced by a human B cell line. FEBS Lett. 1990;272: 133–136. 1699802 10.1016/0014-5793(90)80466-v

[60] Alcami A, Smith GL. A soluble receptor for interleukin-1 beta encoded by vaccinia virus: a novel mechanism of virus modulation of the host response to infection. Cell. 1992;71: 153–167. 1394428 10.1016/0092-8674(92)90274-g

[61] Spriggs MK, Hruby DE, Maliszewski CR, Pickup DJ, Sims JE, Buller RM, et al Vaccinia and cowpox viruses encode a novel secreted interleukin-1-binding protein. Cell. 1992;71: 145–152. 1339315 10.1016/0092-8674(92)90273-f

[62] Gorschluter M, Bohlen H, Hasenclever D, Diehl V, Tesch H. Serum cytokine levels correlate with clinical parameters in Hodgkin's disease. Ann Oncol. 1995;6: 477–482. 7545429 10.1093/oxfordjournals.annonc.a059218

[63] Shin SS, Traweek ST, Ulich TR. Expression of interleukin-1 receptor antagonist protein in malignant lymphomas. An immunohistochemical study. Arch Pathol Lab Med. 1995;119: 247–251. 7887778

[64] Soulillou JP, Douillard JY, Vie H, Harousseau JL, Guenel J, le Mevel-le Pourhiet A, et al Defect in lectin-induced interleukin 2 (IL-2) production by peripheral blood lymphocytes of patients with Hodgkin's disease. Eur J Cancer Clin Oncol. 1985;21: 935–939. 3876220 10.1016/0277-5379(85)90111-7

[65] Zamkoff KW, Reeves WG, Paolozzi FP, Poiesz BJ, Comis RL, Tomar RH. Impaired interleukin regulation of the phytohemagglutinin response in Hodgkin's disease. Clin Immunol Immunopathol. 1985;35: 111–124. 3922653 10.1016/0090-1229(85)90084-4

[66] Mukhopadhyaya R, Advani SH, Gangal SG. Functional evaluation of T-lymphocytes from peripheral blood and spleens in Hodgkin's disease. Br J Cancer. 1987;56: 800–802. 2829954 10.1038/bjc.1987.293 PMC2002398

[67] Damle RN, Tatake RJ, Advani SH, Gangal SG. Affinity of IL-2 receptors and proliferation of mitogen activated lymphocytes in Hodgkin's disease. Br J Cancer. 1990;61:404–406. 2328206 10.1038/bjc.1990.88 PMC1971299

[68] Albert ML, Jegathesan M, Darnell RB. Dendritic cell maturation is required for the cross-tolerization of CD8+ T cells. Nat Immunol. 2001;2: 1010–1017. 11590405 10.1038/ni722

[69] Kanakaraj P, Ngo K, Wu Y, Angulo A, Ghazal P, Harris CA, et al Defective interleukin (IL)-18-mediated natural killer and T helper cell type 1 responses in IL-1 receptor-associated kinase (IRAK)-deficient mice. J Exp Med. 1999;189: 1129–1138. 10190904 10.1084/jem.189.7.1129 PMC2193007

[70] Skeen MJ, Ziegler HK. Activation of gamma delta T cells for production of IFN-gamma is mediated by bacteria via macrophage-derived cytokines IL-1 and IL-12. J Immunol. 1995;154: 5832–5841. 7538532

[71] Ree HJ, Crowley JP, Dinarello CA. Anti-interleukin-1 reactive cells in Hodgkin´s disease. Cancer. 1987;59: 1717–1720. 2435398 10.1002/1097-0142(19870515)59:10<1717::aid-cncr2820591007>3.0.co;2-u

[72] Xerri L, Birg F, Guigou V, Bouabdallah R, Poizot-Martin-I, Hassoun J. In situ expression of the IL-1-α and TNF-α genes by Reed-Sternberg cells in Hodgkin´s disease. Int J Cancer. 1992;50: 689–693. 1312061 10.1002/ijc.2910500504

[73] Steidl C, Connors JM, Gascoyne DR. Molecular pathogenesis of Hodgkin´s lymphoma: increasing evidence of the importance of the microenvironment. J Clin Oncol. 2011;29: 1812–1826. 10.1200/JCO.2010.32.8401 21483001

[74] Liu Y, Sattarzadeh A, Diepstra A, Visser L, van den Berg A. The microenvironment in classical Hodgkin lymphoma: an actively shaped and essential tumor component. Semin Cancer Biol. 2014;24: 15–22. 10.1016/j.semcancer.2013.07.002 23867303

[75] Jona A, Szodoray P, Illes A. Immunologic pathomechanism of Hodgkin´s lymphoma. Exp Hematol. 2013;41: 995–1004. 10.1016/j.exphem.2013.09.014 24099823

[76] Ansell SM, Lesokhin AM, Borrello I, Halwani A, Scott EC, Gutierrez M, et al PD-1 blockade with Nivolumab in relapsed Hodgkin´s Lymphoma. N Engl J Med. 2015;372: 311–319 10.1056/NEJMoa1411087) 25482239 PMC4348009

